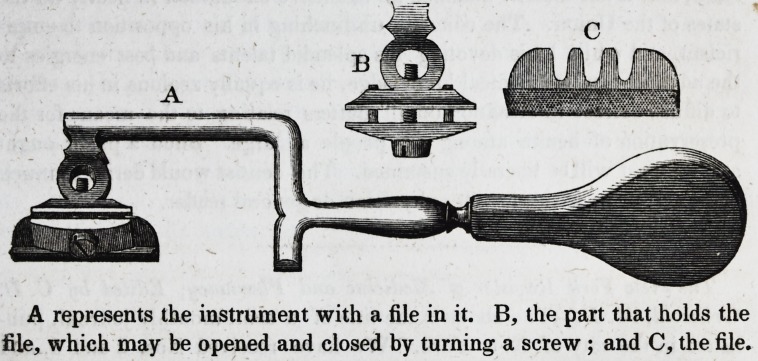# A New File-Carrier

**Published:** 1850-10

**Authors:** 


*A New File-Carrier.*—Some two or three years ago, we published an engraving and description of a file-carrier, invented by Dr. Westcott, which was afterwards improved by Dr. Elliot; and now we announce another, constructed by Dr. Tyrrell, of York, Pa. The annexed cut will convey a better idea of the instrument than any description of it which could be given.

A represents the instrument with a file in it. B, the part that holds the file, which may be opened and closed by turning a screw; and C, the file.

As will be perceived, it is necessary to have files made expressly for the instrument. Although Dr. Tyrrell was kind enough to present us with one of the instruments, we have not, for want of suitable files, had an opportunity of using it. We have no hesitation, however, in pronouncing it to be good.

## Figures and Tables

**Figure f1:**